# Patient Preferences Concerning Humanoid Features in Healthcare Robots

**DOI:** 10.1007/s11948-024-00508-x

**Published:** 2024-10-17

**Authors:** Dane Leigh Gogoshin

**Affiliations:** Practical Philosophy, The RADAR Group, University of Helsinki, Helsinki, FI Finland

**Keywords:** Healthcare robots, Responsible design, Social robotics, Healthcare ethics, Patient preferences

## Abstract

In this paper, I argue that patient preferences concerning human physical attributes associated with race, culture, and gender should be excluded from public healthcare robot design. On one hand, healthcare should be (objective, universal) needs oriented. On the other hand, patient well-being (the aim of healthcare) is, in concrete ways, tied to preferences, as is patient satisfaction (a core WHO value). The shift toward patient-centered healthcare places patient preferences into the spotlight. Accordingly, the design of healthcare technology cannot simply disregard patient preferences, even those which are potentially morally problematic. A method for handling these at the design level is thus imperative. By way of uncontroversial starting points, I argue that the priority of the public healthcare system is the fulfillment of patients’ therapeutic needs, among which certain potentially morally problematic preferences may be counted. There are further ethical considerations, however, which, taken together, suggest that the potential benefits of upholding these preferences are outweighed by the potential harms.

## Introduction

In this paper, I argue that patient preferences for attributes relating to race, culture, and gender should be excluded from the design of robots intended for use in public healthcare settings. While incorporating patient preferences into healthcare robot design should, in principle, enhance patient care – allowing for more individualized and potentially more successful care, enhancing patient self-realization and autonomy – it comes with many ethical risks. I mostly take for granted the moral problematicity of incorporating race, culture, and gender traits into robot design (e.g. bias perpetuation, lack of diversity, inequality^1^), and I limit my focus to public healthcare in a western, culturally diverse, liberal society. It might be the case that these traits are not problematic in certain (e.g. home-based) settings, even within the public system, to the same extent or in the same ways as in public settings. My main focus is therefore tied to the public, clinical setting. However, even home-based practices reflect the values of the healthcare institution and should presumably be consistent with them. By establishing guidelines for responsible healthcare robot design at an early stage, I believe that we can significantly reduce the ethical risks associated with incorporating race, gender, and culture into healthcare robots.

I begin with a set of basic, uncontroversial assumptions about the ends of healthcare, needs, and technology, at a very general level, and steadily narrow the focus to patient preferences concerning particular humanoid features of healthcare robots.^2^ (1) The first assumption is that the aim of healthcare is improved patient well-being.^3^ Patient well-being is a matter of meeting patient needs. Hence the priority of the healthcare system is the meeting of patient needs. Well-being is affected, sometimes dramatically, by preferences and so it is the responsibility of the healthcare system to uphold at least some patient preferences. (2) The second has to do with the nature of care. Patient well-being depends on the well-being of care providers (caregiver and care institution).^4^ (3) The third has to do with the ethical requirements placed on the means (the specific practices) by which these needs are met in virtue of their ethical impact within and outside of the care institution. The practice of care must be consistent with a core set of ethical values and avoid increasing injustice. (1) is in tension with (2) and (3). A *carte blanche* on patient preferences will inevitably interfere with the fulfillment of care provider needs and pose downstream ethical hazards. To solve the challenge and determine whether and the degree to which patient preferences in the context of healthcare robot design should be restricted, I seek a way to balance (1) against (2) and (3).

The paper proceeds as follows. The second section provides important background for the paper and motivates its starting point. The third section argues that only those patient preferences which must be fulfilled to secure therapeutic aims fall within the responsibility of the healthcare system. Via a hypothetical case study, it becomes apparent that such preferences are only knowable *a posteriori*. The fourth section further identifies a significant flexibility in the way that needs are identified and managed, an insight which will factor into later discussion concerning design choices. The fifth section considers a variant of the previous case which involves a potentially morally problematic preference concerning the care provider’s race. This kind of preference puts the care provider between an ethical rock and hard place, since not fulfilling it would interfere with the aims of care and fulfilling it may perpetuate injustice. Further criteria must be introduced in order to reach a defensible decision on such preferences. In the sixth section, observations about the nature of tool design and the place of robots in healthcare shed light onto which features could be legitimate objects of healthcare robot design. The seventh section builds on previous analysis in application to healthcare robot design. The eighth section discusses several ethical considerations which, taken together, favor excluding the traits in question. The paper concludes in the ninth section.

## Starting Points

### Background

As noted by Fosch-Villaronga (2019, ch. 3), there is a lack of standardized evaluation criteria for robots; typically, different assessments are used in each case. This is due to the fact, he writes, that every robot is different and works in different contexts with different requirements. This lacuna motivates his robot impact assessment (ROBIA) module (Fosch-Villaronga, 2019, p. 46), which utilizes a general risk-management strategy. While highly practical and thorough, addressing both ethical and legal risks from the standpoint of every stage of robot design, ROBIA is nonetheless highly context-relative (e.g. relative to the robot’s specific features and uses, the company’s risk tolerance, etc.) and cannot generate general normative guidelines which could apply to healthcare robots more broadly.

For the normative foundation in this paper, I look to van Wynsberghe’s (2016) care-centered value-sensitive design (CCVSD) framework,^5^ which combines a teleological approach to healthcare robots with a set of care-centered ethical values.^6^ Whether to incorporate patient preferences for race, culture, and gender traits into healthcare robot design will be steered according to the aim of care and in accordance with care values. CCVSD is at most a springboard, however, for it does not provide a framework for needs determination, or, relatedly, for determining which patient preferences the healthcare system is responsible for upholding. This paper aims to rectify that lacuna.

### The Aim of Healthcare

The first step in determining the proper role of patient preferences in healthcare robot design is to identify their role in good care. It is therefore important to understand the relationship between good healthcare and patient well-being since, per WHO, healthcare is first and foremost a matter of patient well-being. There are various theories of well-being on offer, each with its own understanding of the relationships between well-being, needs, and preferences.^7^ I would like to stay neutral about these views, but I do hold, minimally, that well-being depends, at least in part, on the fulfillment of needs (Braybrooke, 1987), whatever these needs actually consist in.^8^ I will understand patient well-being, therefore, as a matter of meeting patient needs. I will return to the question of how to conceptualize needs shortly. First, I seek to further refine the relationship between healthcare and patient well-being according to the teleological terms provided by van Wynsberghe (2016), which draws heavily on Joan Tronto’s care ethics (Tronto, 1993, 2010).

As van Wynsberghe points out (2016, p. 22), care is a difficult concept to articulate. It can only really be understood, according to Tronto, in terms of the *practice* of care. Care practice is, in turn, (1) a matter of identifying and meeting needs and (2) manifesting care values through the meeting of these needs. “It is through the manifestation of these values that one comes to understand what care really is in practice” (van Wynsberghe, 2016, p. 24). The needs at stake in the care practice are not just those of the patient, but of the care provider as well. Successfully meeting the varied and dynamic needs of so many counterparts – where some of these needs are only knowable within the care context – is a practical and logistical challenge. Providing a guideline for identifying those needs is a conceptual one. This section attempts to provide such a guideline.

As pointed out by van Wynsberghe (2016, p. 31), identifying these needs is a highly fraught endeavor. Per Fraser (1989), it is “one of the foremost political struggles of any account of care.” This is complicated further by the fact that needs are highly individual, dynamic, and sometimes only become manifest within the care practice itself. Properly identifying them requires therefore both an *a priori* conceptualization as well as the diachronic attention of and interaction with care providers and the environment. How to then actually meet those needs, even once identified, is a further challenge – not just in terms of logistics, but in terms of situating those needs within the context of the needs and resources of their care providers. The successful care of the patient is only possible when the needs of the latter group are also met. However, achieving or managing patient well-being is the aim of care (per WHO), whereas managing care provider well-being is the means. In this light, patient needs are primary. It is now possible to make the following claim.

**C1.** Patient well-being, understood as the fulfillment of patient needs in accordance with care values, though it depends on the well-being of care providers, is the priority of healthcare.

## The Relevance of Patient Preferences

Needs must now be carefully qualified. An obvious starting point is to identify universal (or “basic”) human needs – those needs which must be fulfilled for any human being to attain basic well-being (see e.g. Braybrooke, 1987). Even then, however, we are left with the question of whose responsibility it is for meeting those needs. The domain of responsibility of concern in this paper is that of the public healthcare system. I suggest that the needs for which it is responsible is a function of the degree to which a patient is dependent on it (the extent of their malady, what their treatment plan entails, whether, e.g., they are being treated at home or in hospital, on a full-time or part-time basis). The healthcare institution is responsible, e.g., for the basic social needs of patients who are hospitalized on a full-time basis; it may not be for part-time or at-home patients. In addition to universal human needs, patients have specific therapeutic needs: the need for treatment (or other care solution) that improves their quality of life, expected lifespan or general health (Maertens de Noordhout et al. 2022, p. 15). From this, it may be said that.

**C2**. It is a patient’s therapeutic needs which warrant their entry into the healthcare system and which determine the healthcare system’s priority.^9^

Excluding preferences from the set of patient needs seems an obvious next step until we learn that patient preferences may relate directly to patient well-being.

On one very influential theory of needs (Doyal & Gough, 1991), the two basic, universal needs are physical health and autonomy. In care practice, for which the principle of patient autonomy is a guiding principle, care routines such as bathing can be used in order to develop patient autonomy (van Wynsberghe, 2016, p. 29). “[A]utonomy in care for self-actualization is a matter of developing and choosing rather than doing things oneself (basic skills) or being free to decide on one’s individual life (privacy) (Pols, 2004, p. 61)” (Wynsberghe, 2016, p. 29). We might conclude, on this basis, that autonomy is solely concerned with needs fulfillment. However, according to Pols, upholding a patient’s preferences is key to upholding patient autonomy. Accordingly, we can neither *a priori* exclude the fulfillment of patient preferences from the healthcare system’s domain of responsibility, nor can we, for previously mentioned reasons, take a *carte blanche* approach to it.

Recall that the priority of the healthcare system is the fulfilling of the *therapeutic* needs of the patient. Suppose that a spinal injury patient (who is otherwise a healthy, neurotypical adult in his early 50s), Patient S, can be subjected to significant psychological stress (caused by not being able to uphold his preference for, say, private bathing) without threatening a successful therapeutic outcome (e.g. restoration of motor control). We can say that Patient S’s therapeutic need has been met, even though his overall health and well-being were temporarily negatively impacted by acting against his preference. Should the distress of non-private bathing (not upholding his preference for private bathing) interfere with his potential for therapeutic success – if, e.g., it would render him unwilling or unable to engage in therapy – then his preference should be upheld. I therefore propose to understand those patient preferences the healthcare system ought to assume responsibility for fulfilling, in negative terms – as those which, when not fulfilled, undercut patient well-being. I take it that there is no difference, from the standpoint of how the healthcare system should treat those preferences, between preferences and needs. These two statements can be combined and restated as follows.

**C3**. Patient preferences should be upheld (treated as needs) only when doing so is crucial to successful therapeutic outcomes.

Patient S’s situation gives rise to a further important insight, since his therapeutically-relevant preference was not foreseeable.

**C4**. Which preferences will impact a patient’s particular therapeutic aims and how they will impact these aims is knowable only *a posteriori*.

This insight will inform our approach to patient preferences in healthcare robot design. First, however, we must get some clarity on the right approach to Patient S, once it has been determined that his preference is to be treated as a need. For this, we can further refine our conceptualization of needs on two points. The first concerns the nature of needs and the second concerns the relationship between resource availability and needs.

## Needs Conceptualization

Universal, objective human needs are those needs which must be fulfilled for any human being to attain basic well-being, or so I assume in this paper. In this light, (1) needs are themselves the means to the end of well-being.^10^ And (2) available resources are going to shape those needs as well as the means for attaining them (van Wynsberghe, 2016, p. 34^11^). Consider the need for cleanliness, which is necessary for physical health; physical health is necessary for well-being. Cleanliness should not be pursued for its own sake, detached from its place in well-being. The same is true for activities like eating and exercising. We risk the very things they are meant to bring about – health and well-being – when we treat them as ends in themselves. By eating in a way that is detached from its role in well-being, strictly for pleasure, e.g., we risk eating the wrong kinds and amounts of food and thus our health. How we meet our need for cleanliness, nutrition, fitness, etc. is determined by contextual factors. In the domain of healthcare, it is a matter of the particular patient’s abilities, the care provider’s abilities, and the facilities of the care environment. It might be possible to meet our need for cleanliness in multiple ways: by a traditional shower or bath, with or without assistance, or in-bed wet or dry baths provided by care providers. Our choice of means is a function of our physical abilities (mobility) and the resources (care providers and cleaning facilities/tools) available to us. These observations can be generalized as follows.

**C5**. Needs fulfillment is (merely) instrumental to the end of well-being.^12^

**C6**. There are multiple means, in principle, by which we might satisfy needs, and these means are, at least in part, a function of context and resource availability.

Both C5 and C6 introduce some flexibility to an approach to handling patient needs. Patient needs do not constitute ends in themselves and needs fulfillment is partly determined by available resources. Where to draw the line with respect to optimizing needs-fulfillment, including whether and to what extent a care provider should make new resources available introduces a whole new can of worms, so to speak, and is not something that can be duly addressed from the armchair of speculation. However, as will be discussed in the seventh and eighth section, this flexibility can and should factor into considerations regarding healthcare robot design and implementation.

We return briefly to Patient S. Although there exist many potential means of fulfilling his need for cleanliness (a component of health and well-being), the standard ones significantly negatively impact his psychological well-being and, let us further assume, in a way that impedes his therapeutic success. Patient S is a good candidate for a robotic bathing assistant. Still, he does not, strictly speaking, need one; he needs good hygiene. So if a robotic bathing assistant is unavailable, then he does not, in a strict sense, need *it*. There are potentially other avenues for achieving sufficient hygiene in a way that respects his preference. However, if those avenues are unavailable or unsustainable (due, e.g., to their impact on the care providers), however, then additional resources and new solutions (like robot bathing assistants) may be warranted. In turn, these additional resources affect what needs we can attribute to patients. Should Patient S be in an environment where a robotic bathing assistant is available, e.g., it could be legitimately argued that he needs it. With respect to healthcare robot design choices, we must take into account, as a priority, patient needs as they exist in the context of presently available resources.

Consider next the basic human need for social interaction. When a patient is removed from their social environment and placed into a healthcare institution on a full-time basis, the latter becomes responsible for fulfilling this need. There are multiple means of doing so, in theory. The institution may hire human and animal companions for the patient; they may facilitate social interactions with the patient’s friends, family, and pets. They may rely on their own staff to provide this interaction – either in dedicated moments or in the course of exercising their other care duties to the patient.

Putting the insights of this section together, patient preferences, insofar as they concern objective needs, are subjugated to the aim of well-being and, of most significance to the care system, to the aim of successful therapeutic intervention. In order to identify which preferences are legitimate candidates for inclusion into healthcare robot design, we will need to consider what the proper role of healthcare robots is. First, however, we turn to a morally problematic variant of the case of Patient S.

## Morally Problematic Patient Preferences

Suppose that Patient S, in addition to his preference for private bathing, expresses a strong preference for Asian medical personnel. He happens to be part of an Asian community within a western, English-speaking country and although there are Asian nurses and orderlies at the hospital where he is registered, none of the ones assigned to him are Asian. He refuses their care and his therapy becomes impossible. The care institution must therefore either reject him as a patient or find a way to accommodate his preference. They can hire new Asian staff, reassign existing Asian staff, or utilize, if available, robotic alternatives. Presumably (or so we take for granted in this paper), Patient S’s attitude is morally problematic, but if the care institution can accommodate it without bringing about new ethical harms or compromising the care of other patients, then (existing policy providing), we can reasonably expect it to do so. It is thus not *prima facie* evident that the care institution is off the hook when it comes to upholding Patient S’s morally problematic patient preference. Hence, it is possible to assert the following.

**C7**. The healthcare system may be responsible for upholding some morally problematic patient preferences. Should Patient S be in a care setting where a robotic bather is available and he expresses a preference for this option, then his preference should be upheld. To reiterate a previous point, although Patient S’s need for cleanliness can be met in principle without the robot, meeting it in the standard way negatively impacts his well-being. If a robot is unavailable, he does not technically need it.^13^ However, if no other avenues for achieving it in a way that respects his preference are available or if they are unsustainable, then additional resources and new solutions should be considered.

Note the proper order of things in this context, an observation I will return to later: healthcare technology should be driven by clearly identified and established therapeutic problems and needs and steered by the care provider.^14^

## The Proper Role of Healthcare Robots

In this section, I consider the role that healthcare robots should play in the care system. This is a crucial step in determining the necessary features of healthcare robots, since it is their role which should determine their features. In order for the design (these features) to have normative warrant, the role intended for the robot must itself be justifiable. Morgan et al. (2022) refer generally to healthcare robots as “medical robots,” robotic systems deployed in clinical settings (e.g. service robots, surgical robots, and socially assistive robots). I take it that not all such robotic systems qualify as *social robots*, as defined by Skewes et al. (2019, p. 1),^15^ but the healthcare robots of interest in this paper do meet this definition.

As before, I begin with some uncontroversial assumptions. First, I consider the nature of tool design.^16^

**C8.** Tools are designed and manufactured in order to solve particular problems. They are not meant to provide exclusive means of solving a given problem.

As with all medical technology, healthcare robots should be introduced only when, minimally, they can solve a specific problem in a better way, or with better overall results, than existing resources can provide. As is repeated continually in the literature on healthcare technology, traditional care resources are too limited to address the needs of a growing patient base (van Wynsberghe, 2016, p. 1; WHO, 2023; Hajłasz & Mielczarek, 2020). Healthcare robots, it is argued, can help expand these resources (Kachouie et al., 2014). But accepting this as fact does not itself generate a blanket prescription for which technologies, with which features, should be introduced to solve which problems. This is a matter for care providers to determine on the basis of known problems and existing resources and must be guided by the aim of fulfilling patients’ therapeutic needs. Next, I consider the necessity of distinctly humanoid features in healthcare robots. The features of concern in this paper are those which relate specifically to race, gender, culture, and potentially other aspects of appearance (e.g. age). Legitimizing patient preferences for these features in the healthcare context presupposes that a human being is necessary for fulfilling the related therapeutic need. However, (1) as suggested in the eighth, it is not self-evident that we should rely on the performance of routine care tasks to fulfill patients’ social needs. So there is no *a priori* reason – as far as patients’ social needs are concerned – to make healthcare technology humanoid. Therefore, (2) it is only insofar as the accomplishment of certain tasks – in order for the problem to be solved or optimized by the robot – requires an independently functioning robot (which does not itself require live, proximal human presence), that a humanoid robot (or robot with humanoid features) could, in principle, be necessary. That is to say that it is only in the case that the task requires, e.g., that the patient accept the robot’s use in therapeutic intervention, and that this acceptance is tied to specific human physical features, that a humanoid robot (or a robot with humanoid features) is necessary. Accordingly, we can make the following claim.

**C9**. The extent to which a healthcare technology needs to be humanoid or have humanoid features is a function of the degree to which the tasks it is designed to accomplish require a distinctly social basis and, further, that this basis is specifically humanoid.

We can, presumably, significantly reduce the set of morally problematic preferences when we delimit the need for humanoid features in healthcare robots in this way. Identifying which tasks require humanoid robots (or robots with humanoid features) is clearly an empirical matter. We cannot know *a priori* which healthcare tasks will require humanoid feature-based patient acceptance. Hence, which healthcare tasks require robots with humanoid features is knowable only *a posteriori.*

This means, once again, that empirical research is needed to make responsible design choices. What is more, even if we presuppose that robots with humanoid features are necessary for a given task, we cannot know *which* humanoid features are necessary. It may be possible that most people can relate in the necessary way (for acceptance) to very minimally humanoid features (which are not associated with gender or race). This, however, once again, is strictly an empirical matter. We can now refine the above claim as follows.

**C10**. Which healthcare tasks require robots with humanoid features and which humanoid features are crucial, are strictly empirical matters.

Note that existing empirical studies which confirm that people tend strongly to anthropomorphize robots or do so more when the robots are humanoid (Breazeal, 2003) and, further, tend to assign race, culture, and gender to robots (cf. Bartneck et al., 2018; Bernotat et al., 2017; Eyssel, Friederike, & Hegel 2012; Louine et al., 2018; Perugia et al., 2022; Roesler et al., 2022) do not entail that we require humanoid features or specifically racial or gendered features for social interaction, nonetheless for therapeutic interventions.

## Application to Healthcare Robot Design

In this section, I consider how the results of previous analysis should inform the responsible design of healthcare robots.

**C1.** Patient well-being, understood as the fulfillment of patient needs in accordance with care values, though it depends on the well-being of care providers, is the priority of healthcare.

**C2**. It is a patient’s therapeutic needs which warrant their entry into the healthcare system and which determine the healthcare system’s priority.

**C3**. Patient preferences should be upheld (treated as needs) only when doing so is crucial to successful therapeutic outcomes.

**C4**. Which preferences will impact a patient’s particular therapeutic aims and how they will impact these aims is knowable only *a posteriori*.

**C5**. Needs-fulfillment is (merely) instrumental to the end of well-being.

**C6**. There are multiple means, in principle, by which we might satisfy needs, and these means are, at least in part, a function of context and resource availability.

**C7**. Tools are designed and manufactured in order to solve particular problems. They are not meant to provide exclusive means of solving a given problem.

**C8.** Tools such as healthcare robots are designed and fabricated in order to solve particular problems. They are not meant to provide exclusive means of solving a given problem.

**C9**. The extent to which a healthcare technology needs to be humanoid or have humanoid features is a function of the degree to which the tasks it is designed to accomplish require a distinctly social basis and, further, that this basis is specifically humanoid.

**C10**. Which healthcare tasks require robots with humanoid features and which humanoid features are crucial, are strictly empirical matters.

1. From C1-C3, patients’ therapeutic needs must be the driving concern behind the design and introduction of healthcare technologies. Those and only those patient preferences which must be fulfilled in order to achieve therapeutic aims should be included among these needs (i.e. the healthcare system is responsible only for these particular preferences).

2. From C4, only those patient preferences which are provably (via empirical evidence) necessary (not just sufficient) for therapeutic success should be incorporated into healthcare robot design. We should thus gather statistical data on specific therapy-related patient preferences. Presumably, for the sake of resource optimization (in order to meet the needs of all care parties), only the most statistically significant preferences should be factored into healthcare robot design.^17^

It is important to note that the need for empirical studies is a matter of conceptual importance to the argument of this paper. There are certain decisions concerning robot design that can be made based on values and principles alone. One could presumably make a case against race, culture, and gender (bias-perpetuating) features in robots simply for their moral hazards. It has been argued that things are not so simple, however, given the equal but competing values at stake in good care practice as well as inherent conflicts among the various actors (patients, caregivers, and care institution) within the healthcare system.

There are an increasing number of empirical studies devoted specifically to patient preferences regarding the features of healthcare robots (see Hoppe et al., 2023). I am not trying to (superfluously) make the case that empirical studies concerning patient preferences are important. The crucial point is that the very possibility for determining which patient preferences are relevant to robot design is a wholly *a posteriori* matter and so dependent on empirical data. Every individual patient will potentially have therapy-related preferences which cannot be known *a priori*. The only viable approach to this problem is to gather and generate data on actual therapy-related preferences.

3. From C5-C10, healthcare robots should address proven (empirically and conceptually established) needs (i.e. accomplish tasks) relating directly or indirectly to patients’ well-being. Given the inherent flexibility relating to patient needs and the means for addressing their needs, particularly as concerns the relationship between resource availability and needs fulfillment, there is a unique opportunity at the design stage to influence the practice of healthcare at a fundamental level.

## Ethical Analysis

In this penultimate section, I discuss several normative concerns which, taken together, provide a compelling basis for excluding the traits in question from responsible healthcare robot design.

A per (2) above, there will be significant potential research costs involved in determining which patient preferences concerning human attributes are necessary in healthcare robots. Relevant empirical studies are not a once-and-for-all matter. Innovation will continually shape the scope of robotic therapies available which will, in turn, shape the set of patient preferences-cum-needs. New studies will be necessary when new therapies become available. Second, robots equipped with one set of humanoid features may be unusable with patient groups who have conflicting preferences, thereby reducing their overall usability. The obviously significant resource burden associated with the required research is of more than practical concern. Resource optimization should be guided by a concern for equitable resource distribution. Recall that good healthcare necessitates the fulfillment of patient needs which itself depends on the needs fulfillment of both caregivers and care institution.

A fuller ethical analysis should take into account at least the following considerations: (a) the moral problematicity of incorporating these traits (which, although largely taken for granted in this paper, will shortly be considered independently), (b) the aim of healthcare as a matter of promoting patient well-being, which is itself tied to patient preferences, (c) the link between that aim and the (meeting of) needs of care providers and care institutions, and (d) ensuring that care is administered in ways that are consistent with a set of care values.

In order to fully exclude the morally problematic patient preferences at issue here from responsible healthcare robot design, in potential violation of (b), we would need to provide further, compelling normative justification. Given that upholding these preferences would conflict with (a), (c), and (d) above, such justification is available. Some might argue that (a) is a sufficient reason on its own. But again, patient-centered healthcare, coupled with the teleological normative approach taken in this paper – according to which it is the aim of providing excellent healthcare that generates normative guidelines for care practices, renders (a) insufficient. This is also the case for (c). While meeting the needs of caregivers and care institution are important in their own right and for the sake of providing good care, as previously established, (b) still takes priority. As to (d), among the care values we are charged to uphold – fairness (per CCVSD), patient safety, patient satisfaction, responsiveness to care, human dignity, physical well-being and psychological well-being (per WHO) – there are conflicts, and there are conflicts between these values and (a). Concerning the first kind of conflict, ensuring patient safety, e.g., might temporarily compromise someone’s dignity. Concerning the second kind, not all patient preferences (those of concern in this paper) will even make it to the discussion table. Should they make it into operational healthcare robots, patients whose preferences have been upheld will receive preferential treatment, creating a situation of unfairness and inequality.

These preferences are, moreover, potentially in conflict with human dignity – the patients’ as well as the caregivers’. Prejudices, while inevitable and often beyond the conscious control of their holders, when publicly acted upon, undermine the dignity of both parties – the one manifesting the prejudice and the object of that prejudice. Suppose, e.g., that there are human caregivers available for a given therapeutic task who are rejected by the patient in favor of a robot who meets the patient’s peculiar preferences; this would amount to undermining the value of the caregiver. Should bias-perpetuating preferences get incorporated into the range of available robots, the care institution itself becomes a complicit party to the undermining. Even supposing that the robot population of a given healthcare setting ends up being perfectly diverse (in terms of race, culture, and gender traits) and that all patients might choose from any among them, problems remain. Although bias-perpetuation and equal opportunity will thereby be reduced, they will not be eliminated; it is unlikely that this diversity will be upheld in actual patient choices. It is possible that a majority of patients will share the same preferences, leading as before to issues with supply, equal representation, and equal opportunity.

Finally, recall the earlier point (C6 and (3) above), that the way needs are identified and fulfilled is affected by resource availability. There is an opportunity at the design conception stage to directly shape the very nature of the problems healthcare technologies are designed to solve. Taking this insight on board, along with the above noted conflicts between upholding patient preferences regarding race, culture, and gender (and potentially also e.g. age) and the aim and values of good care, we have good reason to exclude these preferences from healthcare robot design.

## Conclusion

The aim of this paper, to determine whether humanoid features associated with race, culture, and gender should be incorporated into healthcare robot design, was framed in terms of resolving the *prima facie* conflict between (1) the prospective fulfillment of patient preferences for these features (2) meeting the needs of care providers and institution, and (3) the ethics of the healthcare system. First, in light of the primary aim of the healthcare system, it was argued that only those preferences which can be conceived of as therapeutic needs are the responsibility of the healthcare system to fulfill. However, because which preferences qualify as therapeutic needs is knowable only *a posteriori*, it cannot be determined in advance which preferences will be relevant to healthcare technologies. Empirical studies which reveal what percentage of patients have which preferences will therefore be necessary. Statistically insignificant preferences should, from a resource optimization standpoint, be excluded. Resource optimization, as noted, goes well beyond a concern for pure profits; it is a matter of equitable resource distribution, a crucial aspect of the needs fulfillment of the caregivers and care institution.

It was assumed that certain morally problematic (bias-perpetuating) preferences would survive the statistical significance test and so further evaluative criteria were required. It was argued that there is significant flexibility regarding identifying and fulfilling therapeutic needs. This flexibility in turn positively influences the care provider’s ability to meet their own needs. This helps to resolve the tension between (1) and (2) above. Factoring in the moral risks attached to these preferences, the inevitable conflicts between upholding these preferences and core care values like dignity and fairness, the relationship between quality care and resource distribution, and the unique opportunity at the conceptualization stage of healthcare robot design to influence patient needs satisfaction via resource availability, it was concluded that the preferences in question should be excluded.

## Data Availability

N/A.

## Footnotes

1 I independently consider this problematicity in Sect. "Application to Healthcare Robot Design", but see the following for more in-depth coverage: Fosch-Villaronga et al., 2022; Fosch-Villaronga & Poulsen, 2022; Weßel et al., 2021; Sparrow et al. 2020. These authors argue that incorporating race, culture, and gender traits into mass produced technologies (esp. social robots) will significantly perpetuate biases, reduce diversity, promote inequality, etc., in society.

2 My concern is not with specifically humanoid robots. I assume that even non-humanoid robots can be social robots and can be racialized, genderized, and embedded with cultural symbols. I am interested in whether racial, gender, or cultural traits should be included in the design of social, healthcare robots, which I define at the start of Sect. “The Proper Role of Healthcare Robots”.

3 This is derived from the standards of health documented by WHO, n.d.

4 “The needs of the patient mark the starting point of the care process and the process then revolves around a caregiver taking steps to meet these needs. But we can’t forget that the caregiver has needs too: needs in terms of resources, skills, and responsiveness from the care-receiver to understand when needs have been met as well as their own personal needs” (van Wynsberghe, 2016, p. 76).

5 See Friedman et al. (2007) for the VSD approach.

6 CCVSD values: well-being, client choice, privacy, confidentiality, respect for life, truthfulness, and fairness (van Wynsberghe, 2016, p. 25). The WHO framework for people-centered health (per van Wynsberghe 2016, p. 24) values: patient safety, patient satisfaction, responsiveness to care, human dignity, physical well-being and psychological well-being.

7 Simons and Baldwin (2021, p. 990) provide a helpful, basic, and neutral definition of well-being: “Wellbeing is a state of positive feelings and meeting full potential in the world. It can be measured subjectively and objectively using a salutogenic approach.”

8 Van Wynsberghe (2016, p. 32) references the “capabilities approach” of Martha Nussbaum (2000) and Amartya Sen (1993) (needs *qua* capabilities). She further notes (2016, p.34) that needs (per Verbeek, 2011) are partly a function of available resources and that needs are determined by context and practice; accordingly, she suggests, each care practice will be attached to a different set of needs (2016, p. 76). She does not, however, provide a method for needs identification.

9 It is assumed that meeting a patient’s basic needs is a necessary condition of meeting their therapeutic needs.

10 Even on the view that needs satisfaction is constitutive of well-being, the major point here, regarding needs identification, holds.

11 Per Wynsberghe, 2016, p. 34), “[…] needs are often the result of interactions between humans and existing technologies (Verbeek, 2011). For example, the need for surgical intervention is only recognized as a need when we have the technology to provide such an intervention. Without such technologies, the need would be considered a want. It follows then that the introduction of endoscopic tools has introduced a need for “minimally invasive surgery” whereas prior to the technology, conventional surgery was the only option and so conventional surgery was where the need ended.

12 A major criticism of the needs approach concerns whether needs can ever be objective or fundamental, “since claims of need always make tacit reference to some further end for which the thing claimed is necessary […]” (Brock & Miller, 2019).

13 Note that there is a strong limit on the relationship between resource availability and needs. Lack of food does not affect one’s need for food. The way in which I think we should understand Verbeek’s (2011) point about the relationship between needs and resource availability concerns largely (as per C6) the means by which we address needs.

14 It might be that enhancement or augmentation are or become the concern of healthcare providers. In this case, although the line between therapy and enhancement and augmentation might not always be very clear, the scope of patient preferences which the healthcare system is responsible for fulfilling would likely differ from what is proposed in this paper. However, I think that enhancement and augmentation presently largely fall outside of the current objectives of the public healthcare system and so are beyond the scope of this paper to address.

15 As “programmable devices that are designed to act within the physical and symbolic space of human social interactions, and which have affordances for what people common-sensically call social interactions.”

16 We acknowledge the ongoing debate about whether we should view current or future robots as tools (see Gunkel, 2023 for an argument against). In this paper, they are conceived, from a design standpoint, as highly advanced tools meant to solve complex problems.

17 Whether empirical proof is possible is itself a difficult issue. Here, however, are some rough ideas. In addition to gathering actual therapeutic preferences on the basis of patient experience (via patient surveys, records, and provider feedback), I have in mind something like ad hoc patient surveys that address preferences pertaining to the task for which a robot is being considered. For a bathing assistant robot, e.g., bathing-related preferences would be solicited. These surveys could be administered to the wider public, to patients currently receiving care, and to prior patients.
